# Unmet Horizons: Assessing the Challenges in the Treatment of *TP53*-Mutated Acute Myeloid Leukemia

**DOI:** 10.3390/jcm13041082

**Published:** 2024-02-14

**Authors:** Christos Stafylidis, Dimitra Vlachopoulou, Christina-Nefeli Kontandreopoulou, Panagiotis Τ. Diamantopoulos

**Affiliations:** Hematology Unit, First Department of Internal Medicine, Laikon General Hospital, National and Kapodistrian University of Athens, 11527 Athens, Greece; stafi123@hotmail.com (C.S.); dvlachopoulou@gmail.com (D.V.); elina_knt@hotmail.com (C.-N.K.)

**Keywords:** acute myeloid leukemia, *TP53* mutations, *TP53*-mutated acute myeloid leukemia, novel targeted AML therapies, AML immunotherapies

## Abstract

Acute myeloid leukemia (AML) remains a challenging hematologic malignancy. The presence of *TP53* mutations in AML poses a therapeutic challenge, considering that standard treatments face significant setbacks in achieving meaningful responses. There is a pressing need for the development of innovative treatment modalities to overcome resistance to conventional treatments attributable to the unique biology of *TP53*-mutated (*TP53*^mut^) AML. This review underscores the role of *TP53* mutations in AML, examines the current landscape of treatment options, and highlights novel therapeutic approaches, including targeted therapies, combination regimens, and emerging immunotherapies, as well as agents being explored in preclinical studies according to their potential to address the unique hurdles posed by *TP53*^mut^ AML.

## 1. Introduction

P53 is a tumor suppression protein encoded by the *TP53* gene and a vital regulator of genomic stability preservation in response to DNA damage. This regulatory activity occurs through the activation of DNA repair pathways, the triggering of cell-cycle arrest, and the induction of apoptosis [1].

Acute myeloid leukemia (AML) harboring *TP53* mutations, which is now classified as a distinct AML subtype according to the International Consensus Classification (ICC) of myeloid neoplasms and acute leukemias [2], presents a redoubtable clinical challenge as it is associated with an adverse prognosis [3,4,5]. These mutations are observed mostly in treatment-related, relapsed, and elderly AML patients, often characterized by remarkable genomic instability [3,4,5]. While the rate of *TP53* mutations in de novo AML is 5–10%, it is significantly increased (up to 25%) in older patients with de novo AML, with a median age of 60–67 years [6]. Higher frequency rates, up to 35%, are reported in treatment-related AML (t-AML) [6], whereas the highest rates of up to 70% are observed in patients with a complex karyotype and those with loss of chromosome 17/17p, 5/5q, or 7/7q [4,5,7].

Mutated *TP53* induces genomic instability, contributing to leukemogenesis. It also confers unique characteristics to AML and results in the evasion of apoptosis by tumor cells, inherent resistance to conventional chemotherapy, and poor clinical outcomes [1,3,4,5]. Several studies report lower complete response (CR) rates, inferior complete remission duration, and dismal overall survival (OS) among *TP53*-mutated (*TP53*^mut^) AML patients [3,4,5]. Importantly, *TP53* mutations have been found to be predictors of adverse outcomes irrespective of age, chemotherapy regimen, or complex karyotype [3,4]. Moreover, AML patients with *TP53* mutations are at a higher risk of relapse and death after allogeneic stem cell transplantation (aSCT) [8]. Diagnostic approaches such as fluorescence in situ hybridization (FISH), next-generation sequencing (NGS), and in silico approaches may promptly identify these patients and may hold significant predictive value, thus facilitating decision-making relating to treatment strategies [9,10]. Importantly, loss of *TP53*, detected by FISH at diagnosis, has been correlated with poor response to chemotherapy [10].

The challenging management of *TP53*-mutated AML highlights the crucial need for the development of novel therapeutic approaches. In recent years, targeted agents, immunotherapy, and combination strategies have come into the spotlight and have become the subject of intense research in this setting, aiming to overcome the hurdle of the intrinsic treatment resistance caused by *TP53* mutations. In this review, we discuss the role of *TP53* mutations in AML and outcomes based on current treatment options, as well as examining data on innovative agents that are currently being investigated in the preclinical setting and clinical trials.

### 1.1. The Role of TP53 in AML

*TP53* is a 20-kbp tumor suppressor gene located on chromosome 17p13.1 [6]. It encodes for the transcription factor p53 and functions as the “guardian of the human genome” [6]. The p53 protein is a key transcription factor, playing a pivotal role in tumor suppression through DNA repair, cell cycle arrest, differentiation, senescence, apoptosis, autophagy, metabolism, and chemosensitivity [11,12]. The protein contains five important domains: the N-terminal trans-activation domain, a proline-rich domain, a central DNA-binding domain (DBD), a C-terminal oligomerization domain, and a regulatory domain [6].

Since its first description in 1979, *TP53* has been the most frequently mutated gene across all human cancers. It has been previously described that more than 50% of human tumors carry *TP53* mutations, and that many others carrying wild-type *TP53* alleles exhibit decreased *TP53* activity via other mechanisms [13,14]. However, recent data have demonstrated that *TP53* mutations are found in 39% of patients with cancer, a frequency lower than that of previous reports [15]. One of the most well-studied functions of *TP53* is its role in limiting cellular proliferation in response to aberrant oncogene expression. Therefore, *TP53* inactivation through gene deletion or mutation enhances the effect of oncogenes and plays a key role in promoting uncontrolled proliferation of cancer cells. Germline *TP53* mutations cause Li-Fraumeni syndrome (LFS), a disorder that predisposes patients to different types of cancer, including sarcomas, breast cancer, leukemias, and lymphomas [16,17].

It has been observed that the vast majority of de novo AML cases have intact, unaltered *TP53* alleles [18]. However, the frequency of genomic *TP53* alterations is increased in certain patients [4,5,6,7]. In AML, *TP53* mutations are mostly missense somatic substitutions, mostly heterozygous, and include those that are observed in the known hotspot sites of the gene [19]. Diverse genetic aberrations in *TP53*, such as chromosomal alterations leading to allelic gain, loss, or frameshift insertions and deletions have also been described, with the impact ranging from partial to complete loss of function (LOF), mostly in the germline LOF mutations that underlie LFS [19]. Gain of function (GOF) mutations with varied effect sizes are also present in different *TP53* mutants and are thought to mostly result from their binding to different proteins, including transcription factors [20,21]. GOF *TP53* mutants have also been reported to affiliate with epigenetic pathways, e.g., binding and enhancing transcription of the methyl-transferases MLL1 and MLL2 [22]. Monoallelic *TP53* mutations frequently have co-mutations in other genes, mostly *TET2*, *SF3B1*, *ASXL1*, and *DNMT3A,* and are likely to be subclonal events with varying impacts on outcomes of MDS/AML [23]. On the other hand, multihit *TP53*^mut^ MDS/AML represents a distinct disorder, with co-mutations occurring in less than 25% of cases [24]. Finally, the mutational burden of *TP53* has also arisen as a crucial prognostic factor and determinant of therapy choice in AML cases [25]. Despite being one of the most studied genes, *TP53* is still considered “undruggable”, so future studies are needed to ascertain the role of *TP53* mutations in myeloid malignancies.

### 1.2. Current Treatment Options for TP53-Mutated AML

Intensive chemotherapy (IC) with an anthracycline and cytosine arabinoside (AraC) remains the backbone of treatment in patients with newly diagnosed (ND) AML. Eligibility for IC is largely based upon age and comorbidities, hence patients with *TP53*^mut^ AML, who are frequently elderly, may be unfit for this treatment option. Additionally, the presence of *TP53* mutations in AML patients who receive anthracycline- and cytarabine-based induction chemotherapy has been previously associated with inferior outcomes, with reported initial response rates of 20–30% and poor OS of less than a year [4,5]. Baseline *TP53* variant allelic frequency (VAF) has been previously shown to be predictive of response to cytarabine-based treatment, with VAF ≤ 40% being associated with a superior CR/CR with incomplete hematologic recovery (CRi) rate of 79% and a median OS of 7.3 months. This is juxtaposed with VAF > 40%, which has been associated with a CR/CRi rate of 35% and a median OS of 4.7 months [25].

Lower-intensity therapies, including low-dose cytarabine (LDAC) monotherapy or in combination with cladribine and hypomethylating agents (HMAs), are also being used in these patients and are an attractive option since they are accompanied by significantly lower toxicity. There is conflicting data available regarding the efficacy of lower-intensity chemotherapy regimens. A single-center study demonstrated superior CR rates in *TP53*^mut^ AML patients receiving IC as compared to patients treated with lower-intensity regimens (45% vs. 14.3%), but no difference in OS (8.8 months versus 9.4 months respectively) [26]. On the contrary, another study demonstrated lower CR rates among patients with *TP53*^mut^ AML regardless of regimen intensity, and also showed that the intensity of therapy does not predict improved survival [3].

Azacitidine (AZA) and decitabine (DEC) are HMAs that are currently being used either alone or in combination with other agents in the management of *TP53*^mut^ AML. Although the efficacy of AZA monotherapy in AML has been previously demonstrated [27], with a reported CR/CRi rate of 28% [27], its efficacy in AML harboring *TP53* mutations is not well established. A randomized phase three trial comparing the impact of AZA on the survival of AML patients versus conventional care regimens (CCREs), including IC, LDAC, and best supportive care, has shown that the median OS was prolonged in *TP53*^mut^ patients treated with AZA compared to those treated with CCREs (7.2 months versus 2.4 months, respectively); however, this result did not reach statistical significance [28].

Correspondingly, data regarding the efficacy of DEC monotherapy is conflicting [29,30]. A retrospective study has shown similar CR rates among *TP53*^mut^ AML patients treated with either LDAC or a 5-day or 10-day DAC regimen (DEC5 and DEC10, respectively), as well as comparable OS rates among all treatment arms [29]. Accordingly, a study of AML patients treated with DEC has also shown no response or survival benefit in *TP53*^mut^ patients versus *TP53* wild type (*TP53*^wt^) ones [30]. Conversely, a single-institution trial evaluated the efficacy of DEC10 in AML patients and demonstrated exceptionally higher responses in *TP53*^mut^ patients (100% versus 41% in the *TP53*^wt^ arm) [31]. These responses were accompanied by clearance of *TP53*^mut^ leukemic clones in most of the cases, but mutation clearance was never complete [31]. Although *TP53* VAF predicts response and OS in AML patients treated with IC, no effect has been demonstrated on response rates and OS in those treated with HMAs [25]. Moreover, despite the fact that DEC augments chemotherapy responses in *TP53*^mut^ AML, with a currently unknown underlying mechanism, these responses are not durable and do not significantly affect subclones bearing *TP53* mutations [31]. Nonetheless, this enhanced effect paves the way for the design of more combination strategies in these patients.

Recently, the combination of AZA and Venetoclax (VEN), a selective B-cell lymphoma-2 (BCL-2) inhibitor, has become the cornerstone in the treatment of elderly AML patients who are ineligible for IC [32]. First-line treatment of *TP53*^mut^ AML with poor-risk cytogenetics using AZA and VEN initially showed promising results, with a study reporting CR and CRi combined rates of 41% in the combination arm versus 17% in the AZA monotherapy arm, exceeding the historical standards of 28% CR rates [5,33]. However, the duration of response (DOR) and median OS were similar among both treatment arms (6.5 versus 6.7 months and 5.2 versus 4.9 months, respectively) [33]. Furthermore, a study evaluating the efficacy of DEC10 and VEN combination in patients with ND AML has shown pronouncedly inferior outcomes in *TP53*^mut^ patients compared to *TP53*^wt^ patients, with reported ORR and CR/CRi rates in *TP53*^mut^ patients of 66% and 57%, versus 89% and 77%, respectively, in the *TP53*^wt^ group [34]. Importantly, the 60-day mortality rate was higher in *TP53*^mut^ patients (26% versus 4% in *TP53*^wt^), and OS was profoundly lower in these patients (5.2 versus 19.4 months in *TP53*^wt^) [34]. It has been previously demonstrated that *TP53* mutations disrupt the BAX/BAK pathway and establish an elevated activation threshold in leukemic cells (LCs). Although VEN initially suppresses this effect, LCs finally avoid BCL-2 inhibition due to competitive advantage, thus conferring resistance to VEN [35]. Additionally, adaptive resistance associated with alterations in mitochondrial homeostasis and increased oxidative phosphorylation has also been observed [36,37]. Despite this resistance to VEN, its incorporation in novel combination therapies in *TP53*^mut^ AML may still be promising. Concurrent inhibition of BCL-2 and myeloid leukemia 1 (MCL-1) can achieve long-term outcomes by increasing the early apoptotic response in *TP53*-deficient cells, thus making this approach highly promising [36].

ASCT remains the only potentially curative option for high-risk AML patients who are in remission after induction treatment. Prior data have supported that transplanted *TP53*^mut^ patients have yielded superior outcomes as compared to non-transplanted patients who receive other treatment regimens or palliative care [3,25,38]. However, whether aSCT is beneficial for *TP53*^mut^ patients is controversial. A single-center study has shown that although transplanted patients have had significantly superior outcomes in comparison to non-transplanted patients, this association was lost in time-dependent and landmark analysis [39]. Previous studies have also shown poor outcomes in *TP53*^mut^ patients with AML/MDS and AML as compared to *TP53*^wt^ patients, with an increased risk of relapse and death [8,39,40]. A recent meta-analysis has shown a pooled two-year OS of 30% and a pooled relapse rate of 61% at two years post aSCT [41]. Moreover, only a few patients, irrespective of age or performance status, are able to proceed to aSCT at first remission [25,34]. A large multicenter trial (COMMAND) has demonstrated that only 18% of *TP53*^mut^ patients have been bridged to aSCT [42]. The COMMAND trial has also reported a median event-free survival (EFS) and median OS of 12.4 and 24.5 months, respectively [42]. Conversely, a 3-year EFS and OS rate of 61% and 66.3% have been recorded by a retrospective study of *TP53*^mut^ patients post aSCT [43]. Lower *TP53* VAF has been associated with improved clinical outcomes in transplanted patients [25,39]. Hence, VAF may be implemented as a future tool for selecting which *TP53*^mut^ patients may benefit from aSCT. Among other factors, pretransplant minimal residual disease (MRD) and complex karyotype have been associated with inferior outcomes, while the type of pre-aSCT treatment and conditioning regimen have not had a significant impact on clinical outcomes [39,43,44]. Nevertheless, efforts to optimize transplant outcomes by modifying the intensity of conditioning or using novel drug combinations as induction or maintenance treatment are warranted. Notwithstanding the poor outcomes reported, aSCT still remains an appealing choice for achieving long-term survival in *TP53*^mut^ patients.

### 1.3. Currently Available Combination Strategies

Recently, several combination strategies for the management of AML patients harboring *TP53* mutations have been investigated in clinical trials. A recent cohort study evaluated the efficacy of DEC, LDAC, aclarubicin, and granulocyte colony-stimulating factor (G-CSF) [DCAG regimen] versus standard chemotherapy in *TP53*^mut^ AML patients [37], based on the previous encouraging results of a multicenter phase 2 trial which reported 82.4% ORR and 64.7% CR rates relating to the DCAG regimen in elderly AML patients [45]. Although differences were not statistically significant, a trend towards higher ORR, CR, and OS rates was observed in the DCAG arm [37]. Importantly, patients with poor cytogenetics in the DCAG arm displayed superior responses with a significantly higher CR rate of 56.3% and a median OS of 7.8 months (versus a CR of 0% and median OS of 3 months in the standard chemotherapy arm) [37].

The combination of LDAC with clofarabine or cladribine alternating with DEC has been evaluated in the management of treatment-naïve elderly AML patients with reported CR and CRi rates of 59% and 7%, respectively, and a median OS of 12.5 months [46,47,48]. Long-term results from these studies have shown that among all patients, those with *TP53* mutations yielded the lowest responses, with a composite complete remission (cCR) rate of 44% and a poor median OS of 5.4 months [48]. Addition of AZA prior to treatment with high-dose cytarabine (HiDAC) and mitoxantrone, considering that epigenetic priming induced by AZA before cytotoxic chemotherapy could contribute to enhanced responses has been previously examined in a phase 1 study of high-risk AML patients, resulted in an ORR of 61% [49]. However, patients with *TP53* mutations seemed not to benefit from this regimen [49]. A study of *TP53*^mut^ AML patients demonstrated that the combination of DEC, chidamide, a histone deacetylase inhibitor (HDACi) with a priming regimen consisting of omacetaxine mepesuccinate (an alkaloid herbal derivative), cytarabine, and G-CSF (HAG) yielded potent responses with an ORR of 71.4% and manageable toxicity [50]. The study sample size was small, and thus, definite conclusions cannot be drawn; however, these promising results warrant further investigation in the near future [50].

CPX-351, a liposomal formulation of cytarabine and daunorubicin, constitutes the contemporary treatment of AML with myelodysplasia-related changes (MRC-AML) and t-AML [51]. Real-life data from the French cohort study of CPX-351 indicated that *TP53* mutations were the only predictive factor of inferior responses in multivariate analysis, although high-risk molecular prognosis subgroups, including patients with *ASXL1* and *RUNX1* mutations, displayed higher than expected response rates [51]. In accordance, similar results were reported by another retrospective study, demonstrating inferior responses in *TP53*^mut^ patients, who achieved lower CR and CRi rates as compared to *TP53*^wt^ patients (33% versus 62%, respectively) [52]. Consistently, a post hoc analysis of a randomized phase 3 trial showed poor outcomes in *TP53*^mut^ patients [53]. Opposingly, a German retrospective analysis demonstrated that the presence of *TP53* mutations did not impact responses to CPX-351 or survival [54]. Nonetheless, the role of CPX-351 in the management of *TP53*^mut^ AML needs to be further evaluated.

### 1.4. Novel Therapeutic Agents

Considerable progress has also been made regarding the development of novel agents, including mutant p53-targeted approaches and immunotherapy.

#### 1.4.1. Targeted Treatments

Novel targeted therapies incorporated into combination regimens have also been explored in the *TP53*^mut^ AML setting. Pevonedistat (PEVO)–an inhibitor of the NEDD8-activating enzyme (NAE)–seems to exert antiproliferative effects on LCs, and preclinical data supports synergistic effects with AZA and VEN [55,56,57]. A phase 1b study of unfit, treatment-naïve AML patients treated with PEVO and AZA showed improved responses with an ORR of 50%, with *TP53*^mut^ patients achieving a CR and partial response (PR) rate of 80% [55]. Based on these results, a phase 2 study consisting of *TP53*^mut^ AML patients was conducted, but failed to show enhanced CRR rates and was prematurely terminated [56]. Intriguingly, a phase 1/2 study evaluating the efficacy of combined PEVO, AZA, and VEN in ND secondary AML reported a CR/CRi rate of 64%, but a dreadful 1-year OS of 0% in *TP53*^mut^ patients, contrary to a median OS of 18 months in *TP53*^wt^ patients [57]. Moreover, the DOR differed significantly among these patients [57]. These conflicting results may be attributable to the different VAF of patients, since the second study included only *TP53*^mut^ patients with a VAF of >30%. although these results seem discouraging, data are scarce and derived from small studies; thus, PEVO may still have a role to play in this setting.

Ibrutinib, a Bruton tyrosine kinase (BTK) inhibitor has been shown to impede the proliferation of human AML blasts in vitro, either alone or combined with cytarabine or daunorubicin [58]. A randomized phase 2 study evaluated the outcomes of adding ibrutinib to DEC10 versus DEC10 monotherapy in elderly, previously untreated AML patients [59]. Surprisingly, although the addition of ibrutinib did not yield favorable outcomes, *TP53*^mut^ was correlated with higher responses and CR/CRi rates of 56% [59]. However, these responses did not translate into a superior OS [59]. Although ibrutinib’s efficacy in *TP53*^mut^ AML needs to be further validated, it remains a highly appealing approach.

Finally, bortezomib, a proteasome inhibitor, has been widely investigated for use in the management of AML patients, since it has been associated with potent antiproliferative properties [60]. A randomized phase 2 trial of AML patients treated with either combined bortezomib and DEC10 or DEC10 alone failed to demonstrate any advantage of the combination treatment in those with *TP53* mutations [60]. Moreover, the addition of bortezomib conferred no benefit to the study patients overall [60]. Conclusively, the efficacy of targeted therapies remains ambiguous. Further exploration of these agents in *TP53*^mut^ AML through large clinical trials is warranted.

#### 1.4.2. *TP53* Targeting Agents

Although p53 has traditionally been considered undruggable, efforts have been made to overcome this hurdle and have led to the development of a new, small molecule called “p53 reactivation and induction of massive apoptosis” (PRIMA-1) that can reverse the mutant conformation of p53. This molecule induces protein unfolding and restores wild-type functions to mutant p53, such as induction of apoptosis and promotion of cell cycle arrest [61]. Eprenetapopt (EP) or APR-246, a methylated derivative of PRIMA-1 (PRIMA-1^MET^), is a first-in-class agent that binds covalently to cysteine residues in mutant p53 protein [61]. Preclinical studies have demonstrated that EP exerts apoptotic effects on AML cell lines and primary LCs from AML patients in a dose-dependent manner [62]. Noteworthily, the presence of *TP53* mutations did not significantly affect sensitivity to this agent [62]. Subsequent studies have shown significant synergistic cytotoxicity of EP and AZA in *TP53*^mut^ primary cells from MDS/AML patients [63]. Apart from the reported mutant p53 reactivation, preclinical data have also demonstrated that EP results in glutathione depletion and induction of ferroptosis irrespective of *TP53* status, thus indicating a different mechanism of action that leads to p53-independent cell death [64,65].

Recently, EP’s efficacy in combination with AZA has been evaluated in patients with *TP53*^mut^ MDS and AML in two phase 2 studies, one in the USA and another in Europe [66,67]. EP was administered by intravenous infusion at a fixed dose on days 1–4 of each 28-day cycle and AZA was administered subcutaneously, at the standard dose, for seven days of each 28-day cycle [66,67]. *TP53*^mut^ AML patients in the US trial achieved ORR and CR rates of 64% and 36%, respectively, and a median OS of 10.8 months [66]. However, the sample size was small and only patients with oligoblastic AML (20–30% marrow blasts) were included [66]. The European trial, additionally including *TP53*^mut^ AML patients with more than 30% marrow blasts, has demonstrated an ORR of 33% and a CR rate of 17% [67]. However, none of the patients with a high blast count achieved a CR [67]. the median OS in patients with less and more than 30% marrow blasts was 13.9 months and 3.0 months, respectively [67]. Both studies have reported a significant reduction in the *TP53* VAF and p53 expression by immunochemistry in responding patients, with some patients achieving *TP53* negativity (VAF < 5%) [67]. These findings indicate a promising efficacy, since ORR, CR, and OS rates are generally higher than those reported with AZA monotherapy, particularly for patients with oligoblastic AML [67]. Of note, patients with *TP53*^mut^ MDS have also yielded high response rates in both studies, with a CR rate of around 50% [66,67]. The doublet of EP and AZA has also been evaluated in a phase 2 trial of *TP53*^mut^ AML patients, as post-aSCT maintenance therapy administered for up to 12 cycles, with reported relapse-free survival and median OS being 12.5 and 20.6 months, respectively, which is encouraging for this high-risk population [68]. The triplet combination of EP, AZA, and VEN has also been studied recently in the *TP53*^mut^ AML setting. In a phase 1 dose-finding and expansion study, patients with ND *TP53*^mut^ AML achieved an ORR, CR, and CR/CRi rate of 64%, 38%, and 56%, respectively, whereas DOR and median OS were 4.2 and 7.3 months, respectively [69]. Importantly, blast count did not have an impact on patients’ responses [69]. Moreover, *TP53* negativity (VAF < 5%) by NGS was achieved in 27% of patients [69]. These results are highly promising, since the observed CR rates are higher than the CR rates of 22% that have been reported in patients with previously untreated *TP53*^mut^ AML receiving AZA in combination with VEN [69]. Collectively, EP has demonstrated promising efficacy in *TP53*^mut^ AML patients and provides the basis for further investigation in randomized clinical trials in the near future.

#### 1.4.3. Immunotherapeutic Approaches

Interest has also grown regarding the use of immunotherapeutic agents in *TP53*^mut^ AML. CD47 or the “don’t eat me signal” is a transmembrane protein that interacts with signal-regulatory protein alpha (SIRPa), which is expressed in macrophages, and impedes macrophage-mediated phagocytosis [70]. LCs have high levels of CD47, thus escaping immune surveillance [70]. Increased CD47 expression in AML hematopoietic stem cells (HSCs) has been independently correlated with inferior outcomes, thus making the CD47/SIRPa axis an appealing therapeutic target [71]. Blockade of CD47 in AML models has resulted in the induction of phagocytosis and elimination of LCs [71,72]. Magrolimab (MAG) is a novel, first-in-class IgG4 monoclonal antibody against CD47 that acts as a macrophage checkpoint inhibitor and has exerted synergistic effects with AZA and VEN in preclinical in vitro and in vivo studies, with the latter agents eliciting “eat me” signals by upregulating calreticulin [72,73]. A phase 1b study has evaluated the combination of MAG and AZA in patients with previously untreated AML who were ineligible for IC, with the majority of patients (82.8%) having *TP53* mutations [73]. The CR rate was similar among *TP53*^mut^ and *TP53*^wt^ patients (31.9% and 32.2%, respectively), whereas the OS was 9.8 months and 18.9 months, respectively [73]. A phase 1/2 study of the triplet AZA, VEN, and MAG in ND elderly AML, high-risk (HR)-AML, and relapsed/refractory (R/R) AML patients demonstrated an ORR and a CR rate of 74% and 41%, respectively, in ND *TP53*^mut^ patients [74]. Although preliminary results were encouraging, a subsequent phase 3 trial (ENHANCE-2), evaluating MAG and AZA versus physician’s choice of VEN and AZA or IC in *TP53*^mut^ AML was prematurely terminated as MAG failed to demonstrate a survival benefit compared to the current standard of care [75].

Several other agents targeting the disrupted CD47-SIRPa axis are also being explored in MDS/AML. Maplirpacept (MAP) or TTI-622 is a soluble fusion protein with anti-CD47 properties that, unlike other anti-CD47 agents, binds minimally to normal erythrocytes [76]. In vivo studies of AML xenografts have demonstrated the efficacy of TTI-622 in enhancing macrophage-mediated phagocytosis [76]. A phase 1a/1b dose escalation and expansion trial of MAP alone or in combination with other agents in patients with advanced hematologic malignancies, including a cohort of ND *TP53*^mut^ AML patients treated with MAP and AZA, is currently active (NCT03530683). Lemzoparlimab is another anti-CD47 agent that is currently being investigated in patients with HR-MDS and AML, in combination with AZA and/or VEN (NCT04202003, NCT04912063). A recent phase 1b study has evaluated the efficacy of AK117, an anti-CD47 agent, in combination with AZA as a frontline treatment for AML patients and has demonstrated a CR and CR/CRi rate of 45% and 55%, respectively [77]. Evorpacept (EVO) or ALX148 has been associated with increased LC phagocytosis in *TP53*^mut^ AML lines and mouse xenograft models, and its combination with HMA and/or VEN confers better survival [78]. Hence, EVO entered a phase 1/2 trial, which aimed to study its combination with VEN and AZA in patients with AML (ASPEN-05 trial, NCT04755244). However, ASPEN-05 was terminated, based on data from the ASPEN-02 trial, which was also terminated, reporting failure to achieve superior outcomes in MDS patients treated with EVO and AZA [79]. Other anti-CD47 agents that are currently being studied in AML, combined with AZA and VEN include DSP107 (NCT04937166) and SL-172154 (NCT05275439), whereas a phase 1b study (NCT04485052) of IB188 (letaplimab) plus AZA in AML was suspended. Concisely, the employment of anti-CD47 agents in the treatment of *TP53*^mut^ has been met with unsatisfactory results.

T-cell immunoglobulin mucin-3 (TIM-3) is a cell-surface glycoprotein that is constitutively expressed on the surface of certain immune cells, such as the T-cells, and acts as a co-inhibitory receptor [80,81]. When interacting with one of its ligands, such as galectin-9, TIM-3 prompts the inhibition of T-cell responses [80,81]. It has also been demonstrated that TIM-3 is overexpressed in LCs and that TIM-3^+^ AML leukemic stem cells (LSCs) secrete galectin-9 in an autocrine loop that regulates self-renewal of these cells via enhanced NF-κB and β-catenin signaling [80,81]. Hence, antibodies targeting TIM-3 provide a highly appealing therapeutic opportunity. Sabatolimab (SAB) or MBG453 is a humanized, high-affinity IgG4 antibody that targets TIM-3 [82]. A phase 1b study that has evaluated SAB in combination with HMAs in patients with HR-MDS and ND AML displayed promising preliminary results, with ND AML patients exhibiting ORR and CR rates of 40% and 25%, respectively, and a median duration of response of 12.6 months [82]. Importantly, durable responses have been observed in patients with adverse-risk mutations, including *TP53*, indicating that this combination may be effective in the *TP53*^mut^ setting [82]. The addition of VEN is also explored in an ongoing phase 1b trial (NCT03940352), which investigates the combination of SAB and VEN in AML and HR-MDS patients. Furthermore, a phase 2 trial (STIMULUS-AML1, NCT04150029) is currently underway, investigating the combination of SAB, AZA, and VEN in patients with ND AML.

CD123 also serves as an appealing candidate for targeting. CD123 is a component of the interleukin-3 receptor (IL-3R) that plays a multifaceted role in hematopoiesis and immune responses; it stimulates HSC proliferation through activation of the PI3K/MAPK pathway and upregulation of antiapoptotic proteins, and it also participates in the modulation of T-cell responses [83]. CD123 is widely expressed in blasts of AML patients, and its overexpression has been correlated with poor prognosis [84]. In vitro and in vivo studies have demonstrated that a novel CD123 x CD3 dual-affinity retargeting (DART) molecule mediates T-cell activation and proliferation, leading to dose-dependent elimination of AML cell lines and primary AML blasts [83]. Flotetuzumab (FLOT) is a CD123 × CD3 DART antibody that has been evaluated in a phase 1/2 study in R/R AML after primary induction failure or in early relapse, with the reported ORR being 30% [85]. Remarkably, *TP53*^mut^ patients yielded encouraging responses with a CR rate of 47% and a median OS of 10.3 months in responding patients [86]. Currently, early-phase trials are also exploring FLOT in post-transplant relapsed AML (NCT04582864, NCT05506956). Pivekimab sunirine (PVEK) or IMGN632 is a first-in-class antibody-drug conjugate (ADC) with a high affinity for CD123, which has displayed synergy with AZA and/or VEN in preclinical models [87]. An ongoing multicenter phase 1/2 study is investigating PVEK as a triplet with AZA and VEN or in combination with MAG in patients with R/R AML or ND CD123^+^ AML [88]. Preliminary data have shown that treatment with the triplet in R/R AML patients has led to an ORR and a composite CR rate (coCR) rate of 51% and 31%, respectively [87]. However, VEN-naïve patients yielded significantly higher responses than those with prior exposure to VEN [87]. Recent data regarding patients in the ND AML cohort receiving frontline triplet treatment have reported robust responses with a CR and a coCR rate of 52% and 66%, respectively, whereas CR and coCR rates for *TP53*^mut^ patients were 13% and 47%, respectively [89]. Rapid MRD negativity was reported in 73% of patients achieving coCR [89]. Exceptionally, high coCR_MRD_ rates were demonstrated among adverse risk patients, *TP53*^mut^ patients included [89]. Triple combination therapy has been also associated with a manageable safety profile [87,89]. A phase 1 clinical trial of PVEK in combination with fludarabine, high-dose cytarabine (HiDAC), G-CSF, and idarubicin (FLAG-Ida) for frontline treatment of ND adverse-risk AML is ongoing (NCT06034470).

Tagraxofusp (TAG) is a CD123-targeted immunotoxin and has been evaluated as monotherapy in a phase 1 trial of AML and MDS patients, with reported responses being modest [90]. However, recent data have supported that AZA, when combined with TAG, overcomes TAG resistance and restores TAG sensitivity, thus providing a rationale for the combination of these two agents [91]. A phase 1b trial of TAG with AZA and/or VEN in AML and MDS patients is ongoing and preliminary results indicate promising efficacy [92]. Remarkably, *TP53*^mut^ patients have achieved a CR/CRi/morphologic leukemia-free state (MLFS) rate of 54%, with a CR rate of 31% [92]. Early-phase studies include the use of TAG as maintenance therapy for post-transplant AML patients (NCT05233618), for ND secondary AML after previous exposure to HMA (NCT05442216), and in combination with gemtuzumab ozogamicin for R/R AML (NCT05716009). Vibecotamab or XmAb14045, a CD3-CD123 bispecific T-cell engaging (BiTE) antibody, is currently being investigated in the treatment of R/R AML, with preliminary data reporting modest ORR rates of 14% [93]. Vibecotamab has also been associated with cytokine release syndrome (CRS), which is manageable with premedication [93]. Other CD123-targeting agents that are in early clinical development include APV0436, MGD024, and CD123 chimeric antigen receptor T-cell (CAR-T) therapy [94]. In summary, these results suggest that these agents may have a role to play in the treatment of AML patients, *TP53*^mut^ included, and research in this field continues to uncover new insights into potential applications of CD123.

Immune-checkpoint inhibitor-based approaches have also been studied in AML. Ipilimumab, an antibody-targeting cytotoxic T-lymphocyte-associated protein 4 (CTLA-4), has yielded a CR rate of 42% in patients with post-aSCT relapsed AML [95]. Nivolumab (NIVO), a programmed cell death protein 1 (PD-1) inhibitor, has been evaluated as first-line AML therapy in combination with idarubicin and AraC and has yielded encouraging responses in *TP53*^mut^ patients [96]. NIVO has also been studied in R/R AML patients, in combination with AZA, with a modest ORR of 33% and an ORR of 13% in *TP53*^mut^ patients [97]. A recent phase 2 trial of R/R AML patients receiving pembrolizumab, a PD-1 inhibitor, with HiDAC demonstrated promising clinical activity in *TP53*^mut^ patients, reporting a CR rate of 40% [98]. A randomized phase 2 trial of AZA with or without durvalumab (DURV), a PD-L1 inhibitor, as first-line treatment for elderly AML patients failed to show a potential benefit; the addition of DURV did not enhance clinical outcomes, and recorded ORR and OS were similar among both treatment arms [99]. Interestingly, responses were similar between *TP53*^mut^ and *TP53*^wt^ patients (ORR 35% and 34%, respectively) [99]. Nonetheless, the use of CTLA-4, PD1, and PD-L1 inhibitors in AML necessitates further research for strong conclusions to be drawn.

Leukocyte-associated immunoglobulin-like receptor 1 (LAIR-1) is an immune inhibitory receptor which is present on most immune cell subsets and is implicated in immunosuppressive responses [100]. It has been demonstrated that LAIR-1 is highly expressed in AML blasts and LSCs and is responsible for the inhibition of intracellular downstream survival signals and blast proliferation. Its expression is relatively lower in normal HSCs, thus rendering LAIR-1 an ideal anti-leukemic target [100]. NC525 is a humanized monoclonal antibody that binds specifically to AML blasts and LSCs while sparing normal hematopoiesis. It induces apoptosis through a unique signaling pathway, without evidence of immunomodulatory effects on other immune subsets [100]. Furthermore, it has been shown that NC525 displays synergistic activity when combined with AZA and VEN and results in leukemic cell destruction in patients who are refractory to VEN-AZA [100]. A phase 1 trial investigating the safety and tolerability of NC525 in patients with advanced HMs, including R/R AML is underway (NCT05787496).

#### 1.4.4. Other Agents

Murine double minute protein 2 (MDM2) is an E3 ubiquitin ligase that negatively regulates the activity of p53 [101]. MDM2 interacts with p53 and promotes its degradation via ubiquitination [101]. Inhibition of MDM2 mediates antileukemic effects in *TP53*^wt^ AML through an increase in p53 levels [101]. A phase 1/1b study has evaluated the use of idasanutlin (IDASA), an oral MDM2 inhibitor (MDM2i), either alone or in combination with AraC, in unfit for IC patients with R/R or ND AML and has demonstrated a coCR rate of 18.9% and 35.6% in patients receiving monotherapy or combination treatment, respectively [102]. A subsequent randomized, double-blind, phase 3 trial (MIRROS trial), evaluating IDASA combined with AraC or placebo in R/R AML patients, has failed to show an improvement in OS, although the overall remission rate was enhanced by the addition of IDAS [103]. Although MDM2i require wt-p53 to be effective, hence being unable to act directly in *TP53*^mut^ AML, they indirectly induce degradation of MCL-1, which is associated with VEN resistance, thus providing a rationale for the combined use of MDM2i and VEN, even in *TP53*^mut^ patients, in order to overcome VEN resistance [104]. Milademetan, an MDM2i, in combination with LDAC, with or without VEN, has been recently explored in AML with discouraging responses and significant gastrointestinal toxicity [105]. A phase 1b trial of IDASA and VEN in R/R AML patients has shown modest responses, with *TP53* mutations having been associated with unfavorable outcomes [106]. A concern regarding the use of MDM2i is whether they select for the outgrowth of *TP53*^mut^ clones since studies have reported emergent *TP53* mutations in some patients [105,106]. Nevertheless, further studies are needed in order to assess the safety and efficacy of these agents in this setting.

Various agents for *TP53*^mut^ treatment are currently in early clinical development. Arsenic trioxide (ATO) has been shown to inactivate *TP53* by inducing proteasomal degradation of mutant p53 and upregulating *TP53*^wt^ functions [107]. Therefore, it can lead to inactivation of proliferation of LCs and apoptosis promotion. Atorvastatin is a potent destabilizing agent of mutant p53; it has been shown that it effectively induces degradation for conformational or misfolded p53 mutants via inhibition of the mevalonate pathway, with minimal effects on wt-p53 and DNA contact mutants [108]. Collectively, these findings provide insight into exploring arsenic compound-based and statin-based therapies for AML harboring *TP53* mutations. A trial of combined ATO and DEC to treat *TP53*^mut^ AML/MDS (PANDA-T0 trial, NCT03855371) and a pilot trial of atorvastatin in *TP53*^mut^ and *TP53*^wt^ malignancies (NCT03560882) are currently enrolling.

#### 1.4.5. Novel Treatments in *TP53*^mut^ AML: Does a Promising Future Await?

In brief, targeted treatments, including those targeting mutant p53, along with immunotherapeutic agents, have yielded vastly different response rates in *TP53*^mut^ AML patients, as seen in Table 1 [53,54,55,56,57,58,64,65,66,67,71,72,80,84,87,90,94,95,96,97,104]. However, these responses have not translated into a survival benefit; the reported median OS was less than a year in the majority of the studies [53,54,55,56,57,58,64,65,66,67,71,72,80,84,87,90,94,95,96,97,104]. Although these results may be discouraging, they derive mostly from small studies; hence, further study is required and these agents may still hold promise for this challenging clinical setting, particularly in combination with HMAs. As seen in Table 2, several ongoing early-phase trials are investigating the use of novel agents in *TP53*^mut^ AML patients and may offer a promising treatment option for these patients in the near future.

### 1.5. Novel Agents in Preclinical Studies

Despite growth in the understanding of AML pathobiology, therapeutic progress is still inadequate. The requirement for improvement has yielded development of novel drugs targeting various molecularly defined AML entities, including p53-based therapies. Cells with mutant or deleted *TP53* frequently have a defective G1 checkpoint and are more dependent on the G2 checkpoint to repair DNA damage; the G2 checkpoint allows p53-deficient AML cells to repair genetic lesions and continue through the cell cycle. Consistent with this finding, inhibition of kinases involved in the G2 checkpoint, such as aurora kinase A (AURKA) and aurora kinase B (AURKB), has induced mitotic catastrophe and p53-independent cell death in *TP53*^mut^ cancer cells [109]. TP-0903, a small molecule originally developed as an AXL inhibitor, is a multikinase inhibitor with activity against AURKA/B, Chk1/2, and other cell cycle regulators and has activity in models of drug-resistant AML with both WT and mutated TP53 [109]. Xpo7, a putative nuclear/cytoplasmic transporter, was recently identified as a factor necessary for the survival of Trp53-knockout (KO) AML cells with the performance of genome-wide CRISPR-Cas9 screens using Trp53-KO and WT mouse AML cells, indicating a synthetic lethal relationship between *TP53* and XPO7 [110]. *TP53*^mut^ targeted therapy aims to abolish *TP53*^mut^ cancer cells or to rescue p53 mutational inactivation. Pharmacological strategies are directed toward regaining p53^wt^-like conformation and p35^mut^ tumor-suppressive functions, abrogating distinct mechanisms underlying p53^mut^ GOF, and promoting p53^mut^ degradation [111,112,113,114,115,116,117]. On the other hand, dysfunctional p53^wt^ targeted therapy aims to rescue p53^wt^ by addressing various AML-related p53^wt^ inactivating mechanisms. As aforementioned, one such strategy involves MDM2i that disrupts WTp53-MDM2 interactions [101]. Table 3 summarizes the available preclinical studies targeting *TP53*/p53 in AML in vitro and in vivo models. Finally, agents that can help overcome resistance to currently available therapies have also been investigated. Targeting mitochondrial metabolism with novel antimitochondrial agents, including electron transport chain complex inhibitors, pyruvate dehydrogenase inhibitors, and mitochondrial ClpP protease agonists has led to enhanced sensitivity of leukemic cells to combination treatment with VEN and AraC and substantially delayed relapse [118].

## 2. Conclusions

In conclusion, the management of *TP53*^mut^ AML remains a formidable clinical challenge. Current therapeutic approaches yield suboptimal outcomes, thus denoting the urgency for tailored strategies addressing the molecular landscape of *TP53* mutations along with the inherent resistance and aggressive nature of the disease. Although the armamentarium of promising approaches keeps expanding, most novel agents have not met with satisfactory efficacy, with survival rates similar to current treatments. However, these data derive mostly from quite small studies, so strong conclusions cannot be drawn. Among novel treatments, immunotherapeutic agents such as pevonedistat, nivolumab, and flotetuzumab have displayed promising efficacy and warrant rigorous investigation through large clinical trials. Preclinically, agents that target *TP53*/p53 have also yielded encouraging responses, thus necessitating their study in the clinical setting. What is certain is that as we delve deeper into the molecular landscape of AML, the significance of *TP53* mutations becomes increasingly apparent, thus requiring a paradigm shift in our clinical strategies, with hopes of fostering a brighter future for patients with *TP53*^mut^ AML.

## Figures and Tables

**Table 1:** Available current data from studies of novel agents in *TP53*-mutated acute myeloid leukemia.

Agent	Study Type	Regimen	Population	*TP53*^mut^ Patients (*n*)	Response	OS (Months)	Refs
Pevonedistat	Open-label, phase 1B, multicenter	PEVO + AZA	Unfit, untreated AML patients	8	CR/CRi/PR 75%	NR	[55]
	Open-label, phase 2, multicenter	PEVO + AZA	≥60 y, untreated, *TP53*^mut^ AML patients	10	CR/CRi 0%	mOS 6.2 m	[56]
	Phase 1/2 single-center	PEVO + AZA + VEN	Unfit ND secondary AML patients, MDS and CMML patients after failure of HMAs	11	CR/CRi 64%	mOS 8.1 m	[57]
Ibrutinib	Randomized, phase 2, multicenter	Ibrutinib + DEC10 vs. DEC10 monotherapy	Elderly, unfit, untreated AML patients	27	CR/CRi 56% in both arms	Inferior OS compared to *TP53*^wt^ patients	[59]
Bortezomib	Randomized, phase 2, multicenter	Bortezomib + DEC10 vs. DEC10 monotherapy	Elderly, ND AML patients	12 in combination arm and 14 in DEC10 arm	CR 17% in combination arm vs. 21% in DEC10 arm	1-year OS 17% in combination vs. 21% in DEC10 arm	[60]
Eprenetapopt (APR-246)	Open-label, phase 1b/2, multicenter	APR-246 + AZA	≥18 y, *TP53*^mut^, HMA-naïve MDS, MDS/MPN, CMML, oligoblastic (20–30% blasts) AML patients	11	ORR 64% CR 36%	mOS 10.8 m	[66]
	Open-label, phase 2, multicenter	APR-246 + AZA	≥18 y, *TP53*^mut^, HMA-naïve MDS, CMML, oligoblastic and >30% blasts AML patients	18	ORR 33% CR 17% CR 27% in oligoblastic AML CR 0% in AML with >30% blasts	mOS 10.4 m mOS 13.4 m in oligoblastic AML mOS 3 m in AML with >30% blasts	[67]
	Open-label, phase 2, multicenter	APR-246 + AZA as maintenance treatment after HCT	≥18 y, *TP53*^mut^, MDS or AML patients post-HCT	14	NA	mRFS 12.5 m mOS 20.6 m (for all patients)	[68]
	Open-label, phase 1, multicenter	APR-246 + AZA + VEN	≥18 y, *TP53*^mut^, untreated AML patients	43	ORR 64% CR 38% CR/CRi 56%	mOS 7.3 m	[69]
Magrolimab	Open-label, phase 1b, multicenter	MAG + AZA	≥18 y, unfit, untreated AML patients	72	ORR 47% CR 32%	mOS 9.8 m	[73]
	Open-label, phase 1b/2, multicenter	MAG + AZA + VEN	≥18 y, unfit, ND or untreated secondary and VEN-naïve or VEN-exposed R/R AML patients	27 in the ND and untreated secondary AML cohort	ORR 74% CR 86% CR/CRi 63%	1-year OS 53%	[74]
Sabatolimab (MBG453)	Open-label, phase 1b, multicenter	SAB + HMA	Unfit, ND or R/R HMA-naïve AML, high risk HMA-naïve MDS and CMML patients	NR	ORR 53.8% in ND AML patients with at least 1 ELN adverse-risk mutation, including *TP53*	NR	[82]
Flotetuzumab	Open-label, phase 1/2, multicenter	FLOT monotherapy	R/R AML/MDS patients	15 in the R/R AML cohort	ORR 60% CR 47%	mOS 10.3 m	[86]
Pivekimab sunirine (IMGN632)	Open-label, phase 1b/2, multicenter	PVEK + AZA + VEN	ND, CD123^+^ AML patients	19	CR 13% coCR 47%	NR	[89]
Tagraxofusp	Open-label, phase 1b, multicenter	TAG + AZA +/− VEN	Unfit, ND, CD123^+^ AML patients	13	CR 31% CR/CRi/MLFS 54%	mOS 9.5 m mPFS 5.1 m	[92]
Nivolumab	Open-label, phase 1/2, single-center	NIVO + idarubicin + AraC	Fit for IC, ND AML patients	8	CR/CRi/CRp 50% for *TP53*^mut^ patients CR 67% for patients with poor-risk mutation profile, *TP53* included	NR	[96]
	Open-label, phase 2, single-center	NIVO + AZA	R/R AML patients	16	ORR 28%	NR	[97]
Pembrolizumab	Open-label, phase 2, two-center	PEMBRO + HiDAC	R/R AML patients	5	CR 40%	NR	[98]
Durvalumab	Randomized, open-label, phase 2, multicenter	DURV + AZA vs. AZA monotherapy	Elderly, unfit, ND AML patients	21 in the combination arm, 17 in the monotherapy arm	ORR 34% in *TP53*^mut^ AML vs. ORR 33% in *TP53*^wt^ AML for both treatment arms	NR	[99]
Idasanutlin	Open-label, phase 1, multicenter	IDASA + VEN	Unfit, ND sAML or R/R AML patients	10	CR/CRi/CRp 20%	mOS 3.7 m	[106]

PEV: pevonedistat, AZA, azacitidine; AML, acute myeloid leukemia; CR, complete remission; CR, complete remission with incomplete count recovery; PR, partial remission; NR, not reported; mOS, median overall survival; VEN, venetoclax; ND, newly diagnosed; MDS, myelodysplastic syndrome; CMML, chronic myelomonocytic leukemia; HMA, hypomethylating agent; DEC10, 10 days decitabine treatment; MDS/MPN, myelodysplastic syndrome/myeloproliferative neoplasm; ORR, overall response rate; mRFS, median relapse free survival; HCT, hematopoietic cell transplant; NA, not applicable; MAG, Magrolimab; R/R, relapsed/refractory; SAB, sabatolimab; ELN, European Leukemia Net; FLOT, flotetuzumab; PVEK, pivekimab sunirine; coCR, composite complete remission; TAG, tagraxofusp; MLFS, morphologic leukemia-free state; PFS, progression free survival; NIVO, nivolumab; AraC, cytarabine; IC, intensive chemotherapy; CRp, complete remission with incomplete platelet recovery; PEMBRO, pembrolizumab; HiDAC, high dose cytarabine; DURV, durvalumab; IDASA, idasanutlin; sAML, secondary acute myeloid leukemia.

**Table 2:** Ongoing clinical trials of interest in patients with *TP53*-mutated acute myeloid leukemia.

Agent	Target	Regimen	Patient Population	Phase	Identifier
Maplirpacept	CD47	Maplirpacept + AZA	ND *TP53*^mut^ AML	1a/1b	NCT03530683
SL-172154	CD47	SL-172154 + AZA +/− VEN	ND or secondary AML and MDS (*TP53*^mut^ included)	1a/1b	NCT05275439
Sabatolimab (MBG453)	TIM-3	SAB + AZA + VEN	ND unfit for IC AML (*TP53*^mut^ included)	2	NCT04150029
Flotetuzumab	CD123	FLOT monotherapy	Post allo-HCT relapsed CD123+ AML (*TP53*^mut^ included)	1b	NCT05506956
				2	NCT04582864
Pivekimab sunirine (IMGN632)	CD123	PVEK + FLAG-Ida	ND, adverse risk, CD123+ AML (*TP53*^mut^ included)	1	NCT06034470
Tagraxofusp	CD123	TAG + GO	R/R AML (*TP53*^mut^ included)	1	NCT05716009
Nivolumab	PD-1	NIVO + DEC + VEN	ND *TP53*^mut^ AML	1	NCT04277442
NC525	LAIR-1	NC525 monotherapy	R/R AML, MDS, CMML (*TP53*^mut^ included)	1	NCT05787496
Arsenic trioxide	p53	ATO + DEC	*TP53*^mut^ AML/MDS	1	NCT03855371
Atorvastatin	p53	Atorvastatin monotherapy	*TP53*^mut^ AML and solid tumors	1	NCT03560882
MbIL-21 NK cells	Antitumor effects	MbIL-21 NK cells + DEC + fludarabine	R/R AML (*TP53*^mut^ patients included)	1	NCT04220684
ASTX727 (oral DEC and cedazirudine)		ASTX727 + entrectinib	R/R *TP53*^mut^ AML	1	NCT05396859

AZA, azacitidine; ND, newly diagnosed; AML, acute myeloid leukemia; NCT, national clinical trial, VEN, venetoclax; MDS, myelodysplastic syndrome; SAB, sabatolimab; FLOT, flotetuzumab; IC, intensive chemotherapy; allo-HCT, allogeneic-hematopoietic cell transplantation; PVEK, pivekimab sunirine; FLAG-Ida, fludarabine, cytarabine, idarubicin and granulocyte colony-stimulating factor; GO, gemtuzumab ozogamicin; R/R, relapsed/refractory; NIVO, nivolumab; DEC, decitabine; LAIR-1, leukocyte associated immunoglobulin like receptor 1; CMML, chronic myelomonocytic leukemia; ATO, arsenic trioxide; mbIL-21 NK cells, membrane-bound interleukin 21 natural killer cells.

**Table 3:** *TP53*/p53 targeting in preclinical studies.

Compound	Target	Model	Mechanism of Action	Combination with Other Therapy	Ref
Compounds that restore p53 wildtype function					
PK7088	Y220C	Cell lines	Selective induction of caspase 3/7 in p53-Y220C cells and restoration of p53^wt^ conformation	NA	
PhiKan083	Y220C	In silico	BAX nuclear export induction to the mitochondria, and restoration of p53 nontranscriptional apoptosis	NA	[113]
NSC319726 (ZMC1)	R175H, R172H	In silico	Zinc chelator, providing optimal zinc concentration for mut p53-R175H proper folding; induction of ROS formation Restoration of p53^wt^ conformation and activity with MDM2-dependent degradation	NA	[114]
PEITC	R175H	Cell lines	Sensitization of p53^mut^ to proteasome-mediated degradation and further restoration of p53^wt^ conformation and transactivation	NA	[115]
COTI-2	R175H, R273H	Cell lines	Restoration of p53^wt^ activity by targeting and binding to misfolded p53 mutant	NA	[117]
Compounds that induce degradation of mutant p53					
PU-H71 (Zelavespib)	R248W	Molm13 and K562 cells	Induction of cell death in *TP53*^wt^, *TP53*-KO, and *TP53*^mut^ cells	VEN enhanced the killing of both *TP53*^wt^ and *TP53*^mut^ cells by PU-H71	[116]
Compounds with miscellaneous targets					
TP-0903 (Dubermatinib)	Multikinase inhibitor	Cell lines	AURKA/B inhibition in *TP53*^mut^ AML G2/M arrest and apoptosis in *TP53*^mut^ AML cells Chk1/2 inhibition in *TP53*^mut^ AML cells DNA damage response through upregulation of pH2AX	Combination of TP-0903 and DEC is active in vitro demonstrating an additive effect TP-0903/DEC prolongs survival in vivo in a HL-60 xenograft model	[109]
XPO7		Mouse cell lines	*Trp53*-KO cells are vulnerable to XPO7 depletion, while XPO7 functions as a *Trp53*-dependent tumor suppressor in *Trp53*^wt^ AML cells Synthetic lethal relationship between *TP53* and XPO7	NA	[110]
RETRA	mutp53-p73 binding	Mouse cell lines	Increase in the expression level of p73, and release of p73 from the blocking complex with p53^mut^, which produces tumor-suppressor effects similar to the functional reactivation of p53. RETRA is active against tumor cells expressing a variety of p53 mutants and does not affect normal cells.	NA	[111]

p53, protein 53; mut, mutant; BAX, Bcl-2 associated X protein; *TP53*, tumor protein 53; KO, knockout; CHIP, carboxyl terminus of Hsc70 interacting protein; AML, acute myeloid leukemia; AURKA/B, aurora kinase A/B; CHK1/2, checkpoint kinase ½; pH2AX, phosphor-histone h2AX; HL-60; human leukemia cell line 60; XPO7, exportin 7; RETRA, reactivation of transcriptional reporter activity; p73, protein 73; VEN, venetoclax; DEC, decitabine; NA, not applicable.
